# Attenuated C-reactive protein response to infection in newborns and neonates: an age-stratified analysis in children with sepsis

**DOI:** 10.3389/fimmu.2026.1862403

**Published:** 2026-07-09

**Authors:** Zhen-Yu Yao, Li-Ting Huang, Hui-Qiang Cai, Jun Wen, Shang-Rong Ji, Jian-Min Lv

**Affiliations:** 1 School of Basic Medical Sciences, Gansu University of Chinese Medicine, Lanzhou, Gansu, China; 2 Xi’an Children’s Hospital & Affiliated Children Hospital of Xi’an Jiaotong University, Xi’an, Shaanxi, China; 3 MOE Key Laboratory of Cell Activities and Stress Adaptations, School of Life Sciences, Lanzhou University, Lanzhou, Gansu, China; 4 Rehabilitation Science Institute, Shaanxi Provincial Rehabilitation Hospital, Xi’an, Shaanxi, China

**Keywords:** C-reactive protein, infection, neonates, newborns, procalcitonin, sepsis

## Abstract

**Purpose:**

C-reactive protein (CRP) is widely used to guide antibiotic therapy in children; however, a uniform CRP cut-off is commonly applied across all pediatric age groups. This study aimed to investigate whether the CRP response to infection differs by age in children with sepsis.

**Methods:**

This single-center retrospective cross-sectional study included children with a discharge diagnosis of sepsis. Patients were stratified into five age groups according to the recommendations of the International Pediatric Sepsis Consensus Conference: newborn (0 < age ≤ 7 days), neonate (7 < age ≤ 30 days), infant (30 days < age ≤ 2 years), toddler/preschool (2 < age ≤ 6 years), and school age/adolescent (6 < age < 18 years). Demographic characteristics, disease severity, vital signs, laboratory parameters, and microbiological test results were collected. Associations between CRP and other parameters were analyzed within and across age groups using nonparametric methods.

**Results:**

A total of 1,228 patients were included. Newborns and neonates had the highest disease severity (vasoactive infusion: 50.0% and 17.7%; invasive ventilation: 56.5% and 24.2%), yet the lowest CRP levels (median 2.32 mg/L and 5.09 mg/L). In contrast, older children had markedly lower disease severity but 5 to 10-fold higher CRP levels. CRP was positively correlated with procalcitonin (PCT) across all age groups (*P* < 0.01), suggesting preserved responsiveness to infection but a reduced magnitude of CRP elevation in newborns and neonates.

**Conclusion:**

The CRP response to infection is substantially attenuated in newborns and neonates despite greater clinical severity. This age-dependent phenomenon suggests that a single CRP cut-off is inappropriate for all pediatric age groups. Clinicians should interpret CRP levels in very young children with caution, recognizing that even modest elevations may indicate clinically significant infection.

## 1 Introduction

Sepsis is a life-threatening disorder caused by a dysregulated host response to infection, with systemic inflammation and organ dysfunction as its core manifestations (1). Sepsis remains a leading cause of morbidity and mortality in children, particularly in newborns and infants (2). Rapid identification of infection is essential for guiding timely antibiotic therapy and improving clinical outcomes (1, 2). C-reactive protein is one of the most widely used acute-phase reactants in clinical practice. Its concentrations rise sharply in response to infection and inflammation, and it is commonly used to support diagnostic decision-making and to monitor antibiotic treatment (3–5). Despite its widespread use, the diagnostic performance of CRP in pediatric sepsis has varied considerably across studies (6–9).

Age is a potentially important but often overlooked factor. Although age-specific thresholds are routinely applied to vital signs and laboratory variables used to identify pediatric sepsis (10), a single uniform CRP threshold, such as 10 mg/L, is generally used regardless of age. This practice implicitly assumes that the inflammatory response reflected by CRP is comparable across all pediatric age groups. However, the immune system undergoes profound maturation during early life (11). Newborns and neonates have distinct patterns of cytokine production and acute-phase protein synthesis, which may influence the magnitude of CRP elevation during infection (12, 13).

Whether this developmental immaturity translates into an age-dependent difference in CRP response has not been systematically investigated in a large age-stratified pediatric cohort. Therefore, this study aimed to compare CRP levels across five pediatric age groups in children with sepsis. We found that the CRP response was attenuated in newborns and neonates compared with older children, a finding with important clinical implications for interpreting CRP values in these vulnerable populations.

## 2 Methods

### 2.1 Study design and participants

This single-center retrospective cross-sectional study was conducted at the Affiliated Children’s Hospital of Xi’an Jiaotong University, China. The study protocol was approved by the local ethics committee. From January 2018 to October 2020, patients under 18 years old with a discharge diagnosis of sepsis were consecutively enrolled. The diagnosis of sepsis was established according to the definition and criteria of the International Pediatric Sepsis Consensus Conference (10). According to the consensus, sepsis was defined as systemic inflammatory response syndrome (SIRS) in the presence of suspected or proven infection. Patients who met this definition on the day of admission were finally included. Patients with chronic systemic inflammatory diseases, inherited metabolic diseases, hematological diseases, malignancies, known or suspected immunodeficiency, or those receiving immunosuppressive therapy were excluded.

### 2.2 Data collection

All clinical and laboratory data were extracted from medical records on the first day of admission. The following data were collected: demographic characteristics, use of vasoactive infusion or invasive ventilation (as indicators of disease severity), microbiological test results (blood culture or molecular methods), vital signs, and laboratory parameters including blood cell counts, blood chemistry panel, PCT and CRP. Because of the retrospective nature of the study, not all patients underwent every laboratory test. The number of patients with available data for each parameter is indicated in the tables. Several important variables that might influence the core conclusions, such as pre-admission antibiotic use, antenatal corticosteroid exposure, and mode of delivery, were not collected because the relevant data were largely missing or unavailable.

### 2.3 Age groups

According to the recommendations of the International Pediatric Sepsis Consensus Conference (10), patients were divided into five groups: newborn (0 < age ≤ 7 days), neonate (7 < age ≤ 30 days), infant (30 days < age ≤ 2 years), toddler and preschool (2 < age ≤ 6 years), and school age and adolescent (6 < age < 18 years). Patients were classified into two subgroups based on microbiological test results: (1) proven infection was defined as a positive culture of blood or other body fluids, or a positive molecular test for a known pathogen; and (2) suspected infection was defined as negative results in all microbiological tests.

### 2.4 Laboratory measurement

Given the retrospective design, laboratory measurements were not performed prospectively by the investigators. Instead, all laboratory tests were routinely conducted by the central clinical laboratory of the Affiliated Children’s Hospital of Xi’an Jiaotong University, China. Blood samples were collected on the first day of admission. Serum CRP levels were measured using a latex-enhanced immunonephelometric assay on a Mindray BC7500 automated analyzer (Mindray, Shenzhen, China). The assay had a detection range of 0.20 ~ 320.00 mg/L, with a sensitivity of 0.5 mg/L. The intra-assay coefficient of variation (CV) was ≤ 10%, and the inter−assay CV was ≤ 15%. Serum PCT levels were measured using a luciferin−enhanced chemiluminescence immunoassay on an NRM411 automated analyzer (NORMAN Biotech, Nanjing, China), with a detection range of 0.05 ~ 100.00 ng/mL and a sensitivity of 0.05 ng/mL. The intra−assay CV was ≤ 10%, and the inter−assay CV was ≤ 15%. Other laboratory parameters were measured using automated analyzers or standard clinical laboratory protocols in the same laboratory. The laboratory participates in the external quality assessment (EQA) scheme organized by the National Center for Clinical Laboratories (NCCL), China, with satisfactory performance throughout the study period. All measurements were performed by trained laboratory technicians who were blinded to the clinical data.

### 2.5 Statistical analysis

Continuous variables were non-normally distributed according to the Kolmogorov-Smirnov test and are presented as medians with interquartile ranges (IQR). Group comparisons for continuous variables were performed using the Kruskal-Wallis H test or Mann-Whitney U test. Categorical variables were expressed as numbers and percentages, and compared using the chi-square test. Correlations between CRP and PCT were assessed with Spearman’s rank correlation. To examine the robustness of the age−dependent CRP difference in CRP levels, we performed a sensitivity analysis using multiple linear regression. For subgroup analyses stratified by infection status (proven vs. suspected infection), multivariable regression models fully adjusted for all potential confounders were not performed, because of the limited sample sizes in certain subgroups. For multiple comparisons, Bonferroni correction was applied based on the number of comparisons. The adjusted significance level was set at 0.05 divided by the number of comparisons. All statistical analyses were performed using SPSS (version 26.0, IBM Corp., Armonk, NY, USA).

## 3 Results

### 3.1 Participant characteristics and disease severity

A total of 1,228 children with a discharge diagnosis of sepsis were included. The number of patients in each age group was as follows (**Table 1**): newborn (N = 186), neonate (N = 62), infant (N = 485), toddler and preschool (N = 351), and school age and adolescent (N = 144). Sex distribution was similar across age groups. We did not systematically assess all types of organ dysfunction because several required clinical details or variables were unavailable in the medical records. However, we recorded two clinically robust indicators of severe organ dysfunction: use of vasoactive infusion, reflecting cardiovascular dysfunction, and use of invasive mechanical ventilation, reflecting respiratory failure. These indicators differed substantially among age groups: newborns and neonates had the highest proportions of patients requiring vasoactive infusion (50.0% and 17.7%, respectively) and invasive ventilation (56.5% and 24.2%). In contrast, the corresponding proportions in older groups were ≤ 4.3% and ≤ 4.9%, respectively (**Table 1**). Vital signs and most laboratory parameters, including white blood cells, platelets, creatinine, and bilirubin, showed the expected age-dependent patterns (**Tables 1**, **2**).

**Table 1:** Population characteristics of all enrolled patients with a discharge diagnosis of sepsis (N = 1,228).

Characteristic	Newborn (N = 186)	Neonate (N = 62)	Infant (N = 485)	Toddler and preschool (N = 351)	School age and adolescent (N = 144)	*P*
Basic characteristic						
Age, months	0.03 ~ 0.23	0.27 ~ 1	1.03 ~ 24	25 ~ 72	73 ~ 174	
Male, %	62.4	58.1	59.2	52.4	53.5	0.142
Weight, Kg	2.71 (2.11 ~ 3.16)	3.06 (2.40 ~ 3.79)	9.00 (6.55 ~ 11.00)	15.00 (14.00 ~ 17.00)	26.00 (23.00 ~ 32.00)	
Disease severity						
Vasoactive infusion, %	50.0	17.7	4.3	0.3	0	**< 0.001**
Invasive ventilation, %	56.5	24.2	4.9	0	0.7	**< 0.001**
Vital signs						
Pulse rate, Beats/minute	145 (132 ~ 152)	146 (140 ~ 155)	128 (120 ~ 135)	106 (98 ~ 116)	94 (88 ~ 100)	**< 0.001**
Respiratory rate, Breaths/minute	55 (48 ~ 61)	49 (43 ~ 55)	32 (28 ~ 38)	25 (23 ~ 28)	23 (21 ~ 24)	**< 0.001**
Mean arterial pressure, mm Hg	50 (40 ~ 55)	54 (50 ~ 59)	60 (59 ~ 63)	67 (64 ~ 70)	71 (70 ~ 73)	**< 0.001**

*P* values less than 0.05 are shown in bold.

**Table 2:** Laboratory parameters for blood tests.

Laboratory parameters	Newborn	Neonate	Infant	Toddler and preschool	School age and adolescent	*P*
White blood cells, ×10^9^/L	11.38 (7.92 ~ 17.51) N=185	11.38 (8.49 ~ 17.49) N=61	10.74 (6.99 ~ 15.55) N=482	8.92 (5.97 ~ 14.36) N=344	7.47 (4.27 ~ 13.10) N=142	**< 0.001**
Platelets, ×10^9^/L	211 (150 ~ 259) N=185	309 (199 ~ 425) N=61	335 (252 ~ 436) N=482	280 (215 ~ 364) N=344	236 (172 ~ 298) N=142	**< 0.001**
Total bilirubin, μmol/L	140.0 (81.8 ~ 205.9) N=175	107.0 (35.1 ~ 184.5) N=60	5.6 (4.0 ~ 8.7) N=476	6.6 (5.1 ~ 9.0) N=345	8.3 (6.3 ~ 11.6) N=138	**< 0.001**
Creatinine, μmol/L	60 (45 ~ 74) N=174	32 (25 ~ 39) N=59	22 (18 ~ 25) N=475	29 (26 ~ 32) N=344	39 (34 ~ 45) N=138	**< 0.001**
Procalcitonin, ng/mL	3.37 (0.66 ~ 9.62) N=174	0.22 (0.09 ~ 0.70) N=59	0.24 (0.09 ~ 0.95) N=472	0.19 (0.06 ~ 0.72) N=337	0.14 (0.06 ~ 0.66) N=136	**< 0.001**
C-reactive protein, mg/L	2.32 (0.52 ~ 8.74) N=181	5.09 (0.99 ~ 27.15) N=59	19.61 (4.06 ~ 54.97) N=471	19.43 (4.43 ~ 47.86) N=341	21.60 (2.98 ~ 66.98) N=139	**< 0.001**

N denotes the actual number of detected patients.

*P* values less than 0.05 are shown in bold.

### 3.2 Pathogen distribution across age groups

On admission, all patients routinely underwent blood culture, and cultures of other body fluids were performed when clinically indicated. Molecular tests, such as PCR and antigen-based assays, were additionally performed according to clinical suspicion, such as suspected viral or mycoplasma infection. The results of pathogen identification were collected, and the distribution of pathogens across age groups is summarized (**Supplementary Table 1**). Overall, after excluding 17 censored cases, 305 of the 1,211 evaluable patients had at least one positive microbiological test. The overall positivity rate was 25.2%. Among these patients, 217 cases (71.1%) were classified as bacterial infections, 13 cases (4.3%) as fungal infections, 41 cases (13.4%) as viral infections, 16 cases (5.2%) as mycoplasma, chlamydia or ureaplasma infections, and 18 cases (5.9%) as polymicrobial infections involving two or more types of pathogens. Bacterial infections predominated across all age groups. Coagulase-negative *Staphylococci* were the most frequently identified organisms. *Escherichia coli* and *Enterococcus faecium* were more commonly detected in younger age groups. *Stenotrophomonas maltophilia*, *Staphylococcus aureus*, *Streptococcus* and *Mycoplasmas* species were more frequently observed in older children.

### 3.3 CRP levels across age groups

**Figure 1:**
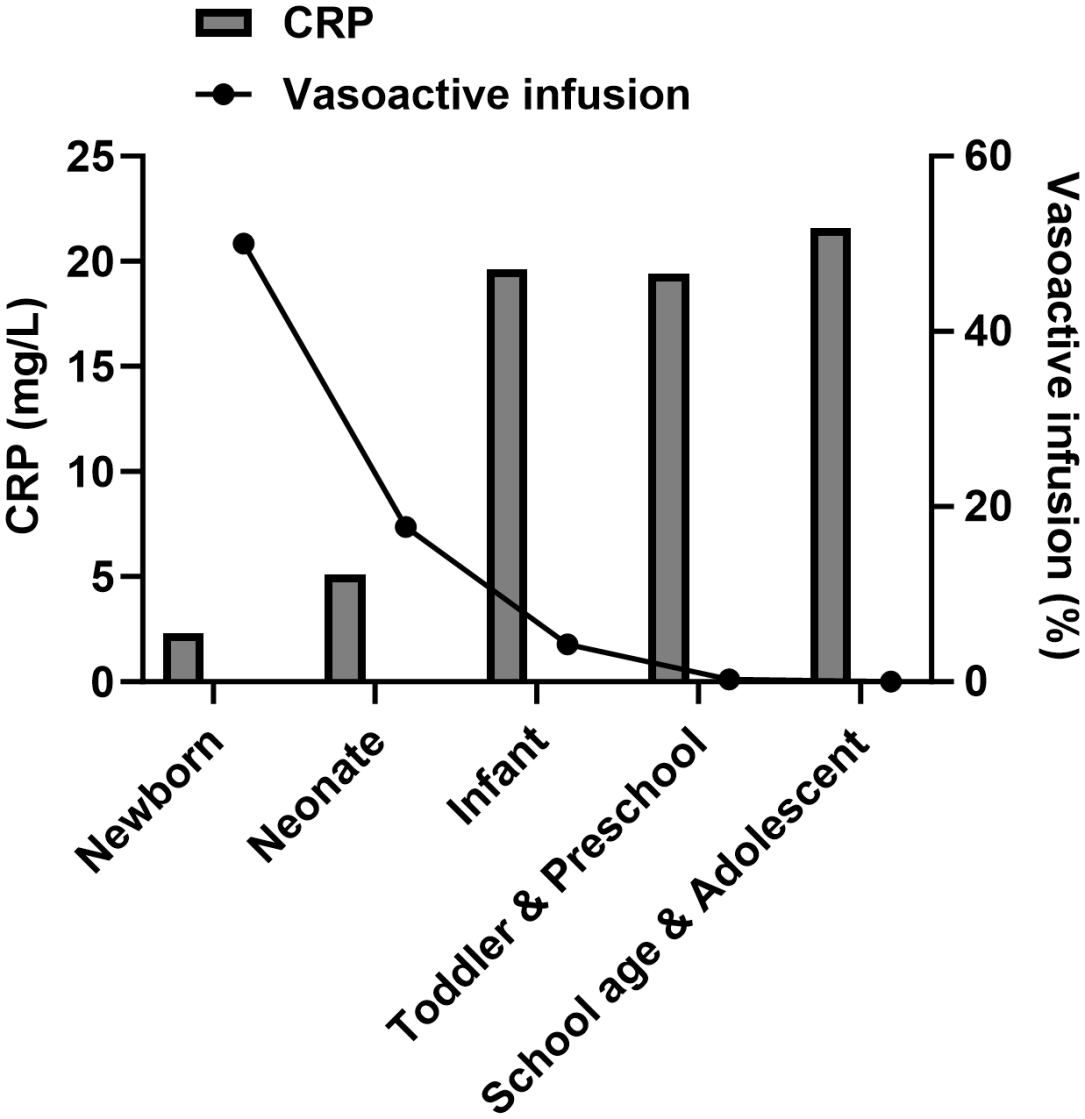
Age-dependent dissociation between CRP level and disease severity. Bars represent median CRP levels (mg/L, left Y-axis); the line with circles represents the proportion of patients receiving vasoactive infusion (%, right Y-axis). Newborns and neonates exhibited the highest disease severity but the lowest CRP levels, contrasting with older age groups. A similar age-dependent pattern was observed for invasive ventilation. Interquartile ranges and sample sizes for CRP are presented in **Table 2**. The distribution of individual CRP levels was presented in **Supplementary Figure 1**. The percentage of vasoactive infusion and invasive ventilation and their sample size are presented in **Table 1**.

CRP levels showed a striking age-dependent pattern. Newborns and neonates had the lowest CRP levels, with median values of 2.32 mg/L and 5.09 mg/L, respectively. In contrast, older children exhibited substantially higher CRP levels, with median values ranging from 19.43 to 21.60 mg/L (*P* < 0.001; **Table 2**; **Supplementary Figure 1**), representing an approximately 5- to 10-fold difference. The low CRP levels in newborns and neonates contrasted sharply with their greater disease severity, suggesting a dissociation between clinical status and CRP response (**Figure 1**). To determine whether the age−dependent differences in CRP levels were independent of potential confounders, we performed a series of analyses.

#### 3.3.1 Influence of prematurity and gestational age on CRP levels

Upon reviewing the enrolled patients, we found that both the newborn and neonate groups included a proportion of preterm patients (**Supplementary Table 2**). Previous study has reported that preterm newborns tend to have lower CRP levels (14). This suggests that prematurity may be an important confounding factor contributing to the lower CRP level observed in the newborn and neonate groups. Within the newborn group, CRP showed weak positive association with gestational age (r = 0.217, N = 181, *P* = 0.004, 95% CI: 0.066 ~ 0.358) and birth weight (r = 0.238, N = 181, *P* = 0.001, 95% CI: 0.091 ~ 0.374), suggesting that preterm patients have slightly lower CRP levels. However, after adjustment for sex, vasoactive infusion and invasive ventilation, multivariable linear regression within the newborn group showed that neither gestational age (β = 0.010, 95% CI: -0.037 ~ 0.057, *P* = 0.678) nor birth weight (β = 0.000, 95% CI: 0.000 ~ 0.000, *P* = 0.148) was a significant predictor of LogCRP (**Supplementary Table 3**). To directly assess the potential confounding effect of prematurity, we restricted the analysis to term patients only. After excluding preterm patients, term newborns and term neonates were combined into a single group. This combined group of term newborns and term neonates (0 ~ 1 month, N = 134) had a median CRP level of 2.91 mg/L (IQR: 0.99 ~ 15.16, N = 134), which was significantly lower than that of term infants, whose median level was 19.63 mg/L (IQR: 4.19 ~ 54.99, N = 461; *P* < 0.001). We also explored whether gestational age within the term range influenced CRP levels. CRP levels were compared between early-term (37 ~ 38 weeks of gestation, N = 29) and full-term (39 ~ 41 weeks of gestation, N = 67) newborns. No significant difference was observed between the two groups (median 1.91 vs. 3.30 mg/L, median difference: -0.990, 95% CI: -2.580 ~ 0.260, *P* = 0.134), suggesting that a 1− to 2-week difference in gestational age within the term range does not substantially affect CRP responses. Taken together, these evidences demonstrate that the attenuated CRP response in newborns and neonates is unlikely to be explained by prematurity.

#### 3.3.2 Influence of onset-to-sampling interval on CRP levels

One concern is that newborns may be sampled earlier in clinical practice, before CRP reaches its peak, which could partly account for their lower CRP levels. Therefore, we examined the interval from symptom onset to admission blood sampling (onset−to−sampling interval). The median onset−to−sampling interval was 2 days (IQR: 1 ~ 4) in newborns, 1 day (IQR: 1 ~ 3) in neonates, 3 days (IQR: 1.25 ~ 5.75) in infants, 3 days (IQR: 2 ~ 6) in toddlers and preschool children, and 4 days (IQR: 2 ~ 7) in school-age children and adolescents (**Supplementary Table 4**). Newborns and neonates had significantly shorter onset-to-sampling intervals than infants and older children (*P* < 0.001). To assess whether the shorter onset−to−sampling interval in newborns could explain their lower CRP levels, we still excluded preterm patients and combined term patients from the newborn and neonate groups. In the combined group of term newborns and neonates, the onset−to−sampling interval was not significantly correlated with CRP levels (Spearman’s correlation = 0.118, N = 134, *P* = 0.174, 95% CI: -0.058 ~ 0.287). After adjustment for onset−to−sampling interval, sex, vasoactive infusion, and invasive ventilation, age group (term newborn and neonate vs. term infant) remained a highly significant predictor of LogCRP (β = 0.455, 95% CI: 0.281 ~ 0.629, *P* < 0.001). In contrast, the onset−to−sampling interval itself had a very small negative coefficient (β = -0.019, 95% CI: -0.037 ~ -0.002, *P* = 0.025), which was opposite to the direction expected if earlier sampling were responsible for the lower CRP levels (**Supplementary Table 5**). After stimulation, CRP levels rise rapidly and peak around 48 hours (15). Therefore, we restricted the analysis to patients with identical onset−to−sampling intervals of 2 ~ 3 days. The results showed that term newborns and neonates (median 3.97 mg/L, IQR: 1.47 ~ 16.95, N = 33) still had markedly lower CRP levels than infants (median 17.54 mg/L, IQR: 5.60 ~ 51.45, N = 95, *P* < 0.01). These findings demonstrate that the attenuated CRP response in newborns and neonates is not explained by differences in onset−to−sampling intervals.

#### 3.3.3 CRP levels according to infection confirmation status

To investigate whether the low CRP levels observed in newborns and neonates were influenced by infection confirmation status, we stratified patients into two subgroups: proven infection and suspected infection. As in the preceding analysis, preterm patients were excluded and term newborns and neonates were combined. We first compared disease severity indicators, including vasoactive infusion and invasive ventilation, as well as vital signs, including pulse rate, respiratory rate, and mean arterial pressure, between the proven and suspected infection subgroups within each age stratum (**Supplementary Tables 6**, **7**). After adjustment, no significant differences in disease severity or vital signs were observed between the proven and suspected infection subgroups in most age strata, except for the rate of vasoactive infusion and pulse rate in term infants. These findings suggest that the proven and suspected infection subgroups were broadly comparable. We then compared CRP levels across age groups separately within the proven and suspected infection subgroups (**Supplementary Table 8**). In both subgroups, term newborns and neonates had the lowest median CRP values (proven infection: 5.38 mg/L; suspected infection: 2.46 mg/L). In the proven infection subgroup, the overall Kruskal-Wallis test showed a significant difference in CRP levels across age groups (*P* = 0.036). Pairwise comparisons with Bonferroni correction showed that term newborns and neonates had significantly lower CRP levels than school-age children and adolescents (adjusted *P* = 0.024), whereas the differences compared with term infants and toddlers/preschool children did not reach statistical significance after correction (**Supplementary Table 9**). Nevertheless, the median difference and 95% confidence intervals showed an age-dependent increase in CRP levels, suggesting that the lack of statistical significance in some comparisons was likely related to the small sample size of the term newborn and neonate group in the proven infection subgroup (N = 26). In the suspected infection subgroup, which included larger sample sizes, term newborns and neonates had significantly lower CRP levels than all older age groups even after Bonferroni correction (all adjusted *P* < 0.001; **Supplementary Table 9**). These results demonstrate that the attenuated CRP response in newborns and neonates is not explained by infection confirmation status. Regardless of whether infection was proven or suspected, newborns and neonates consistently exhibit the lowest CRP levels. Because of the limited sample sizes in proven and suspected infection subgroups, multivariable regression models fully adjusted for all potential confounders were not performed within the two subgroups.

#### 3.3.4 Multivariable linear regression including all age groups

To determine whether the age-dependent difference in CRP levels persisted after adjustment for potential confounders, we performed a multivariable linear regression including all age groups, with newborns as reference. Onset-to-sampling interval, sex, invasive ventilation, and vasoactive infusion were included as covariates (**Table 3**). After adjustment, infants, toddlers/preschool children, and school−age children/adolescents had significantly higher LogCRP than newborns (all *P* < 0.001). The neonate group did not reach significance but showed a trend toward higher CRP levels compared with newborns (β = 0.219, *P* = 0.07), likely owing to the smaller sample size in this group. These findings confirm that age is an independent predictor of CRP levels and that the attenuated CRP response in newborns is not explained by the measured confounders. But we noticed that the model explained only 10.8% of the total variance in CRP levels, with an adjusted R^2^ of 0.108. This suggests that numerous additional factors influencing CRP levels have not been included in this model.

**Table 3:** Multivariable linear regression analysis of factors associated with Log^CRP^ across all age groups.

Variable	β (unstandardized)	95% CI	β (standardized)	*P*
(Constant)	0.635	0.464 ~ 0.807		**< 0.001**
Neonate	0.219	-0.018 ~ 0.457	0.065	0.07
Infant	0.586	0.418 ~ 0.754	0.345	**< 0.001**
Toddler and preschool	0.531	0.346 ~ 0.715	0.278	**< 0.001**
School age and adolescent	0.593	0.376 ~ 0.809	0.228	**< 0.001**
Onset-to-sampling interval (Days)	-0.012	-0.022 ~ -0.002	-0.073	**0.022**
Sex (Female vs. Male)	-0.043	-0.146 ~ 0.059	-0.026	0.407
Invasive ventilation (No vs. Yes)	-0.052	-0.275 ~ 0.170	-0.023	0.645
Vasoactive drug (No vs. Yes)	-0.157	-0.383 ~ 0.069	-0.065	0.173

Dependent variable: LogCRP. Newborns served as the reference group. Model included all age groups as dummy variables, plus onset−to−sampling interval, sex, invasive ventilation, and vasoactive infusion. Adjusted R² = 0.108, N = 913.

*P* values less than 0.05 are shown in bold.

### 3.4 Correlation of CRP with PCT

To determine whether the low CRP levels observed in newborns reflected a complete failure to respond to inflammation or, instead, a preserved but attenuated responsiveness, we analyzed the correlation between CRP and PCT across age groups. CRP was positively correlated with PCT in all age groups, with Spearman correlation coefficients ranging from 0.346 to 0.447 (all *P* < 0.01; **Table 4**). Notably, despite having the lowest CRP levels, newborns still showed a significant positive correlation between CRP and PCT (r = 0.346, *P* < 0.001). This suggests that newborns retain the ability to mount a CRP response to inflammation, although the magnitude of this response is attenuated compared with older children. To assess whether this correlation was robust across different infection confirmation statuses, a subgroup analysis was performed to compare the correlation between CRP and PCT in patients with proven and suspected infections (**Table 5**). In newborns, the correlation was significant in both subgroups. In neonates, the correlation was positive but did not reach statistical significance (r = 0.315, *P* = 0.164; r = 0.324, *P* = 0.057), possibly because of the smaller sample size. Significant positive correlations were also observed in both subgroups among the other age groups. The consistent positive correlation between CRP and PCT across all age groups suggests that newborns retain the ability to mount a CRP response to inflammation. However, the lower correlation coefficient in newborns compared with older children, together with the markedly lower CRP levels, indicates an attenuated CRP response.

**Table 4:** Correlation between CRP and PCT within all age groups.

Statistics	Newborn	Neonate	Infant	Toddler and preschool	School age and adolescent
N	171	56	460	328	132
Spearman Correlation	0.346	0.351	0.447	0.444	0.438
95% CI	0.203 ~ 0.475	0.089 ~ 0.567	0.368 ~ 0.519	0.350 ~ 0.529	0.284 ~ 0.570
*P*	**< 0.001**	**0.008**	**< 0.001**	**< 0.001**	**< 0.001**

N denotes the actual number of detected patients.

*P* values less than 0.05 are shown in bold.

**Table 5:** Correlation between CRP and PCT in proven and suspected infection subgroups.

Age groups	Proven infection			Suspected infection		
	Correlation	95% CI	*P*	Correlation	95% CI	*P*
Newborn	0.446 (N = 34)	0.117 ~ 0.687	**0.008**	0.324 (N = 136)	0.160 ~ 0.471	**< 0.001**
Neonate	0.315 (N = 21)	-0.148 ~ 0.665	0.164	0.324 (N = 35)	-0.020 ~ 0.600	0.057
Infant	0.463 (N = 114)	0.300 ~ 0.600	**< 0.001**	0.437 (N = 344)	0.345 ~ 0.521	**< 0.001**
Toddler and preschool	0.445 (N = 74)	0.235 ~ 0.616	**< 0.001**	0.435 (N = 250)	0.325 ~ 0.533	**< 0.001**
School age and adolescent	0.476 (N = 40)	0.184 ~ 0.691	**0.002**	0.374 (N = 90)	0.175 ~ 0.544	**< 0.001**

N denotes the actual number of detected patients. *P* values are presented without adjustment for multiple comparisons.

*P* values less than 0.05 are shown in bold.

## 4 Discussion

This study provides a systematic, age-stratified description of CRP response in a large cohort of children with sepsis. The key finding is a marked age-dependent dissociation: newborns and neonates had the highest disease severity but the lowest CRP levels, whereas older children had higher CRP levels despite lower disease severity. The robustness of this age-dependent pattern was supported by multiple sensitivity analyses. Newborns consistently showed the lowest CRP levels after exclusion of preterm newborns and neonates, after matching for onset-to-sampling intervals of 2 ~ 3 days, and after stratification by infection confirmation status, including both proven and suspected infection. Moreover, multivariable regression including all age groups confirmed that age was an independent predictor of CRP levels after adjustment for sex, onset-to-sampling interval, invasive ventilation, and vasoactive infusion. However, the low adjusted R² indicates that CRP, as an acute-phase biomarker, is influenced by numerous complex factors, many of which could not be measured or controlled in this multivariable regression model. This is not unexpected, as CRP levels in children can be modulated by factors affecting gene expression, protein synthesis, circulating kinetics, disease progression, and other aspects, all of which may contribute to inter-individual variation. In fact, the multivariable regression model was intended primarily to assess the independent effect of age, rather than to function as a comprehensive predictive tool. Therefore, caution is warranted when interpreting the model’s explanatory power.

Because of the limited sample sizes in several pathogen subgroups, the present study was not sufficiently powered to adequately assess the potential confounding effect of pathogen type on CRP responses. Nevertheless, we summarized CRP levels across pathogen subgroups, including bacterial, viral, fungal, mycoplasma/chlamydia/ureaplasma, and polymicrobial infections, within each age group (**Supplementary Table 10**). In the bacterial infection subgroup, CRP levels differed significantly across age groups (Kruskal-Wallis *P* = 0.028). Pairwise comparisons with Bonferroni correction showed that newborns had significantly lower CRP levels than school-age children and adolescents (median 8.87 vs. 60.99 mg/L; adjusted *P* = 0.02), whereas no significant differences were observed in the other pairwise comparisons. Further analyses were not feasible because of the limited sample size. Future studies with larger sample sizes and appropriate designs are anticipated to thoroughly assess whether pathogen type modifies the age−dependent CRP response. Several potential confounders could not be adjusted for because of the retrospective design, including pre-admission antibiotic use, antenatal corticosteroids exposure, delivery mode, and systematic assessment of multiple organ dysfunction. Pre-admission antibiotics may lower CRP levels and reduce the likelihood of positive cultures, potentially leading to underestimation of the true CRP response. However, such bias would be expected to attenuate rather than exaggerate our main finding. Although pre-admission treatment may reduce CRP levels, newborns still exhibited the highest disease severity. Future prospective studies should systematically record these variables to allow appropriate adjustment.

To determine whether the low CRP levels observed in newborns and neonates reflect a complete failure to respond to inflammation or an attenuated responsiveness, we introduced PCT as a reference biomarker and analyzed the correlation between CRP and PCT across age groups. CRP was positively correlated with PCT in all age groups, although the correlation did not reach statistical significance in neonates. Analyses stratified by infection status further confirmed the robustness of this correlation. These findings are consistent with a physiologically attenuated CRP response. The biological plausibility of an attenuated CRP response in newborns is supported by converging evidence from immunology, hepatology, and developmental biology. The fetus and newborn face a complex set of immunological demands, which shape a distinct neonatal immune system (11). During fetal development, excessive pro-inflammatory responses, particularly TNF-α and Th1-mediated immunity, are downregulated (13). This restrained inflammatory phenotype protects the pregnancy from immune rejection and premature termination. Immediately after birth, the neonatal intestinal tract also downregulates inflammatory responses to endotoxin, thereby avoiding excessive and potentially harmful inflammation during initial microbial colonization (13). Newborns tend to adopt a disease-tolerance strategy that limits inflammatory injury, partly through metabolic regulation of cytokine production. Accordingly, newborns and neonates exhibit cytokine profiles distinct from those of older children and adults (12, 13). This tolerance strategy may be an evolutionary adaptation to prevent excessive inflammation during the transition to an extrauterine environment, but it carries the trade-off of a less pronounced biomarker signal during sepsis (16).

Hepatic maturation may also contribute to the attenuated CRP response. CRP is mainly synthesized by hepatocytes in response to inflammatory cytokine stimulation. During the fetal-to-neonatal transition, the liver undergoes substantial structural and functional maturation, including changes in hepatic blood flow, metabolic activity, transcriptional regulation, and acute-phase protein synthesis. These developmental features may plausibly limit the magnitude and duration of CRP production in newborns and neonates. Previous study has reported that the neonatal liver differs markedly from the adult liver in its hepatic cellular composition (17). This distinct cellular environment, together with the unique cytokine milieu of early life, may contribute to a reduced CRP response during infection. Studies in rats have shown that several hepatic proteins, including acute-phase proteins, have lower serum concentrations before weaning than in adulthood (18). Their concentrations then increased steadily and approached adult levels after weaning (18, 19). This temporal pattern is consistent with our observation that CRP responses appear to mature progressively during infancy. Mackey et al. demonstrated that the transcription factor CCAAT enhancer-binding protein α (C/EBPα) is required for the induction of acute-phase protein in newborn mice in response to lipopolysaccharide. C/EBPα knock-out hepatocytes fail to activate STAT3 in response to IL-6, and show reduced IL-6Rα protein levels partly because of decreased protein stability (20). Although these experimental findings cannot be directly extrapolated to our clinical cohort, they provide a biologically plausible framework for understanding why newborns may have a reduced capacity for CRP synthesis. Further mechanistic studies are needed to determine whether similar pathways contribute to the age-dependent CRP response observed in pediatric sepsis.

Previous study has reported ethnic differences in baseline CRP levels (21). Nevertheless, the physiological phenomenon of low CRP levels in healthy newborns has been observed across multiple populations. Ishibashi et al. found that lower gestational age and birth weight were associated with a significantly smaller increase in CRP levels (22). This observation was subsequently validated in an independent Caucasian cohort (14), which showed that healthy term newborns can mount a CRP response, whereas preterm infants exhibit an even lower and shorter CRP response. Together, these findings suggest that attenuated CRP response in newborns and neonates is not specific to any particular ethnic group.

The clinical implications of our findings are substantial. Currently, a single uniform CRP threshold, for example 5 ~ 10 mg/L, is applied across all pediatric age groups. This practice implicitly assumes that the inflammatory response reflected by CRP is similar at all ages. Our results suggest that interpretation of CRP using a uniform cut-off may be misleading. A CRP value of 5 ~ 10 mg/L, which might be considered only mildly elevated in an older child, may represent a clinically meaningful inflammatory response in a newborn or neonate. Therefore, clinicians should interpret CRP levels in an age-sensitive manner, particularly in the first month of life. In newborns and neonates, even modest CRP elevations should prompt careful consideration of severe infection, especially when accompanied by compatible clinical signs or other markers of disease severity. We are unable to provide specific diagnostic cut-off values for each age group because the current dataset included only children with sepsis and is therefore not suitable for receiver operating characteristic analysis. However, we provide a percentile table of CRP levels for each age group (**Supplementary Table 11**), which may serve as a reference for interpreting a given CRP value in the context of age−specific distributions.

Future prospective, multi−center studies are needed to validate the attenuated CRP response observed in newborns and neonates. Such studies should include controlled assessment of confounding factors and appropriate non-septic reference populations. One import goal is to establish age-specific thresholds for distinguishing septic from non-septic children, thereby improving diagnostic accuracy and supporting more precise antibiotic stewardship. Additionally, mechanistic studies are warranted to elucidate the molecular basis of the attenuated CRP response. In particular, investigation of age-related differences in serum IL-6 levels, downstream signaling pathway, and the epigenetic regulation of acute-phase proteins expression in neonatal hepatocytes may provide import insights into age-specific inflammatory responses and improve the recognition of neonatal sepsis.

## 5 Limitations

Several limitations should be acknowledged. First, this was a single-center retrospective study, which may be subject to selection bias and limit generalizability of the findings. Prospective multicenter studies are therefore needed to validate our results and minimize bias. Second, not all patients underwent all laboratory tests, resulting in variable sample sizes across parameters. However, we reported the actual numbers of patients available for each analysis to ensure transparency. Third, patients were enrolled based on discharge diagnosis, and laboratory data were obtained on the day of admission. Therefore, these data may not have captured the peak inflammation response in all patients. Fourth, pre-admission treatments, such as outpatient antibiotics or antipyretics, may have influenced laboratory parameters at admission and potentially led to an underestimation of the true inflammatory response. As discussed above, this potential bias would likely attenuate rather than exaggerate our main finding. Fifth, because of limited sample sizes in certain specific subgroups, we were unable to perform multivariable regression with complete and simultaneous adjustment for all confounders in all subgroup analyses. Therefore, the subgroup results should be interpreted with caution. Future studies with larger sample sizes are needed to enable more comprehensive adjustment for all potential confounders.

## 6 Conclusion

In this large age-stratified cohort of children with sepsis, newborns and neonates exhibited substantially lower CRP levels than older children despite greater clinical severity. This attenuated CRP response persisted after adjustment for measured confounders and was supported by multiple sensitivity analyses. These findings suggest that CRP interpretation in pediatric sepsis should be age-sensitive. In newborns and neonates, even modest CRP elevations may be clinically meaningful and should not be dismissed.

## Data Availability

The raw data supporting the conclusion of this article are not publicly available due to concerns regarding patient privacy, but are available from the corresponding author upon reasonable request.

## References

[1] Singer M Deutschman CS Seymour CW Shankar-Hari M Annane D Bauer M . The third international consensus definitions for sepsis and septic shock (Sepsis-3). JAMA. (2016) 315:801–10. doi: 10.1001/jama.2016.0287 26903338 PMC4968574

[2] Kawasaki T . Update on pediatric sepsis: a review. J Intensive Care. (2017) 5:47. doi: 10.1186/s40560-017-0240-1 28729906 PMC5518149

[3] Butler CC Gillespie D White P Bates J Lowe R Thomas-Jones E . C-reactive protein testing to guide antibiotic prescribing for COPD exacerbations. N Engl J Med. (2019) 381:111–20. doi: 10.1056/NEJMoa1803185 31291514

[4] Keitel K Samaka J Masimba J Temba H Said Z Kagoro F . Safety and efficacy of C-reactive protein-guided antibiotic use to treat acute respiratory infections in Tanzanian children: a planned subgroup analysis of a randomized controlled noninferiority trial evaluating a novel electronic clinical decision algorithm (EPOCT). Clin Infect Dis. (2019) 69:1926–34. doi: 10.1093/cid/ciz080 30715250

[5] Molloy EJ Strunk T . Role of C-reactive protein for late-onset neonatal sepsis. JAMA Pediatr. (2021) 175:100–1. doi: 10.1001/jamapediatrics.2020.2126 32804193

[6] Brown JVE Meader N Wright K Cleminson J McGuire W . Assessment of C-reactive protein diagnostic test accuracy for late-onset infection in newborn infants: a systematic review and meta-analysis. JAMA Pediatr. (2020) 174:260–8. doi: 10.1001/jamapediatrics.2019.5669 32011640 PMC7042944

[7] Rey C Los Arcos M Concha A Medina A Prieto S Martinez P . Procalcitonin and C-reactive protein as markers of systemic inflammatory response syndrome severity in critically ill children. Intensive Care Med. (2007) 33:477–84. doi: 10.1007/s00134-006-0509-7 17260130

[8] Castelli GP Pognani C Meisner M Stuani A Bellomi D Sgarbi L . Procalcitonin and C-reactive protein during systemic inflammatory response syndrome, sepsis and organ dysfunction. Crit Care. (2004) 8:R234–42. doi: 10.1186/cc2877 15312223 PMC522844

[9] Boonkasidecha S Panburana J Chansakulporn S Benjasuwantep B Kongsomboon K . An optimal cut-off point of serum C-reactive protein in prediction of neonatal sepsis. J Med Assoc Thai. (2013) 96:S65–70. 23724458

[10] Goldstein B Giroir B Randolph A International Consensus Conference on Pediatric S . International pediatric sepsis consensus conference: definitions for sepsis and organ dysfunction in pediatrics. Pediatr Crit Care Med. (2005) 6:2–8. doi: 10.1097/01.PCC.0000149131.72248.E6 15636651

[11] Simon AK Hollander GA McMichael A . Evolution of the immune system in humans from infancy to old age. Proc Biol Sci. (2015) 282:20143085. doi: 10.1098/rspb.2014.3085 26702035 PMC4707740

[12] Donald K Finlay BB . Early-life interactions between the microbiota and immune system: impact on immune system development and atopic disease. Nat Rev Immunol. (2023) 23:735–48. doi: 10.1038/s41577-023-00874-w 37138015

[13] Levy O . Innate immunity of the newborn: basic mechanisms and clinical correlates. Nat Rev Immunol. (2007) 7:379–90. doi: 10.1038/nri2075 17457344

[14] Chiesa C Natale F Pascone R Osborn JF Pacifico L Bonci E . C reactive protein and procalcitonin: reference intervals for preterm and term newborns during the early neonatal period. Clin Chim Acta. (2011) 412:1053–9. doi: 10.1016/j.cca.2011.02.020 21338596

[15] Pepys MB Hirschfield GM . C-reactive protein: a critical update. J Clin Invest. (2003) 111:1805–12. doi: 10.1172/JCI18921 12813013 PMC161431

[16] Kollmann TR Kampmann B Mazmanian SK Marchant A Levy O . Protecting the newborn and young infant from infectious diseases: lessons from immune ontogeny. Immunity. (2017) 46:350–63. doi: 10.1016/j.immuni.2017.03.009 28329702

[17] Nakagaki BN Mafra K de Carvalho E Lopes ME Carvalho-Gontijo R de Castro-Oliveira HM . Immune and metabolic shifts during neonatal development reprogram liver identity and function. J Hepatol. (2018) 69:1294–307. doi: 10.1016/j.jhep.2018.08.018 30171870

[18] Thomas T Schreiber G . Acute-phase response of plasma protein synthesis during experimental inflammation in neonatal rats. Inflammation. (1985) 9:1–7. doi: 10.1007/BF00915406 2579907

[19] Burrin DG Davis TA Fiorotto ML Reeds PJ . Hepatic protein synthesis in suckling rats: effects of stage of development and fasting. Pediatr Res. (1992) 31:247–52. doi: 10.1203/00006450-199203000-00010 1373233

[20] Mackey SL Darlington GJ . CCAAT enhancer-binding protein alpha is required for interleukin-6 receptor alpha signaling in newborn hepatocytes. J Biol Chem. (2004) 279:16206–13. doi: 10.1074/jbc.M400737200 14960573

[21] Oldroyd JC Heald A Bansal N Vyas A Siddals K Gibson M . Inflammatory markers and growth in South Asian and European origin infants in Britain: the Manchester Children's Growth and Vascular Health Study. Atherosclerosis. (2009) 207:227–31. doi: 10.1016/j.atherosclerosis.2009.03.045 19439300

[22] Ishibashi M Takemura Y Ishida H Watanabe K Kawai T . C-reactive protein kinetics in newborns: application of a high-sensitivity analytic method in its determination. Clin Chem. (2002) 48:1103–6. doi: 10.1093/clinchem/48.7.1103 12089183

