# Supplementary material for: Attenuated C-reactive protein response to infection in newborns and neonates: an age-stratified analysis in children with sepsis
Source: Front Immunol. 2026 Jul 9;17:1862403. doi: 10.3389/fimmu.2026.1862403 (PMC13391386; doi:10.3389/fimmu.2026.1862403)
Supplement: Supplementary file 1 [file DataSheet1.docx]

**Table S1 Detailed pathogen distribution by age group.**

| **Age group** | **Bacteria** | **Virus** | **Fungus** | **Mycoplasma / Others** |
| --- | --- | --- | --- | --- |
| **Newborn**  **(N=184, Positive=38)** | Coagulase-Negative *Staphylococci* (9), *Escherichia coli* (7), *Enterococcus faecium* (5), *Klebsiella pneumoniae* (2), *Listeria monocytogenes* (2), *Enterobacter cloacae* (2), *Sphingomonas sanguinis* (1), Others (3) | **/** | *Candida* (2), *Cyberlindnera fabianii* (1), Others (5) | *Ureaplasma spp.* (3), Others (1) |
| **Neonate**  **(N=61, Positive=23)** | Coagulase-Negative *Staphylococci* (5), *Klebsiella pneumoniae* (4), *Escherichia coli* (3), *Enterococcus faecium* (3), *Mycobacterium tuberculosis* (1), *Acinetobacter baumannii* (1), *Serratia marcescens* (1), *Stenotrophomonas maltophilia* (1), Others (1) | *Respiratory Syncytial Virus* (1), *Cytomegalovirus* (1), | Others (3) | *Ureaplasma spp.* (2), *Mycoplasmas* (1), *Chlamydia* (1), Others (2) |
| **Infant**  **(N=480, Positive=119)** | Coagulase-Negative *Staphylococci* (27), *Enterococcus faecium* (14), *Stenotrophomonas maltophilia* (11), *Staphylococcus aureus* (11), *Streptococcus* (7), *Escherichia coli* (3), *Salmonella* (3), *Acinetobacter baumannii* (3), *Bordetella pertussis* (2), *Achromobacter xylosoxidans* (2), *Haemophilus influenzae* (2), *Lactobacillus* (2), *Micrococcus luteus* (2), *Enterobacter cloacae* (2), *Listeria monocytogenes* (1), *Klebsiella pneumoniae* (1), *Branhamella catarrhalis* (1), *Acinetobacter pittii* (1), *Pseudomonas aeruginosa* (1), *C. pseudodiphtheriticum* (1), *Bacillus subtilis* (1), *Leuconostoc pseudomesenteroides* (1), Others (6) | *Epstein-Barr virus* (7), *Coxsackievirus* A (1), *Influenza Virus* (4), *Parainfluenza Virus* (7), *Adenovirus* (1), *Rotavirus* (3), *Norovirus* (2), *Respiratory Syncytial Virus* (2), *Cytomegalovirus* (2), Others (2) | *Candida* (1), *Others* (1) | *Mycoplasmas* (8), Others (9) |
| **Toddler and Preschool**  **(N=344, Positive=80)** | Coagulase-Negative *Staphylococci* (13), *Streptococcus* (7), *Stenotrophomonas maltophilia* (6), *Staphylococcus aureus* (3), *Pannonibacter phragmitetus* (3), *Pseudomonas aeruginosa* (2), *Chryseomicrobium aureum* (2), *Enterococcus faecium* (2), *Micrococcus luteus* (1), *Achromobacter xylosoxidans* (1), *Klebsiella pneumoniae* (1), *Bacillus cereus* (1), *Acinetobacter baumannii* (1), *Salmonella* (1), *Listeria monocytogenes* (1), *Haemophilus influenzae* (1), *Helicobacter pylori* (1), Others (9) | *Epstein-Barr virus* (7), *Influenza Virus* (2), *Parainfluenza Virus* (4), *Adenovirus* (3), *Respiratory Syncytial Virus* (2), *Herpes simplex virus* (1), *Coxsackievirus* (1), Others (2) | *Candida* (2) | *Mycoplasmas* (3), Others (9) |
| **School age and Adolescent**  **(N=142, Positive=45)** | *Staphylococcus aureus* (10), *Streptococcus* (11), Coagulase-Negative *Staphylococci* (2), *Salmonella* (1), *Haemophilus influenzae* (1), *Achromobacter xylosoxidans* (1), *Micrococcus luteus* (1), *Enterobacter cloacae* (1), Others (6) | *Epstein-Barr virus* (4), *Influenza Virus* (3), *Coxsackievirus* (2) | *Candida* (1) | *Mycoplasmas* (6), Others (1) |

**Note:** Numbers in parentheses are isolate counts. One patient may contribute multiple isolates. Therefore, the sum of isolates may exceed the number of positive patients.

**Table S1 Continued**

“Others” in “Bacteria” include reports such as “Gram‑positive cocci”, “Gram‑negative rods”, “mixed growth”, or those without specific species. “Others” in “Virus” and “Fungus” include reports without specific type. “Others” in “Mycoplasma / Others” include infectious markers (e.g., LPS, 1,3-β-D-glucan and so on).

“/” indicates no isolate in that category.


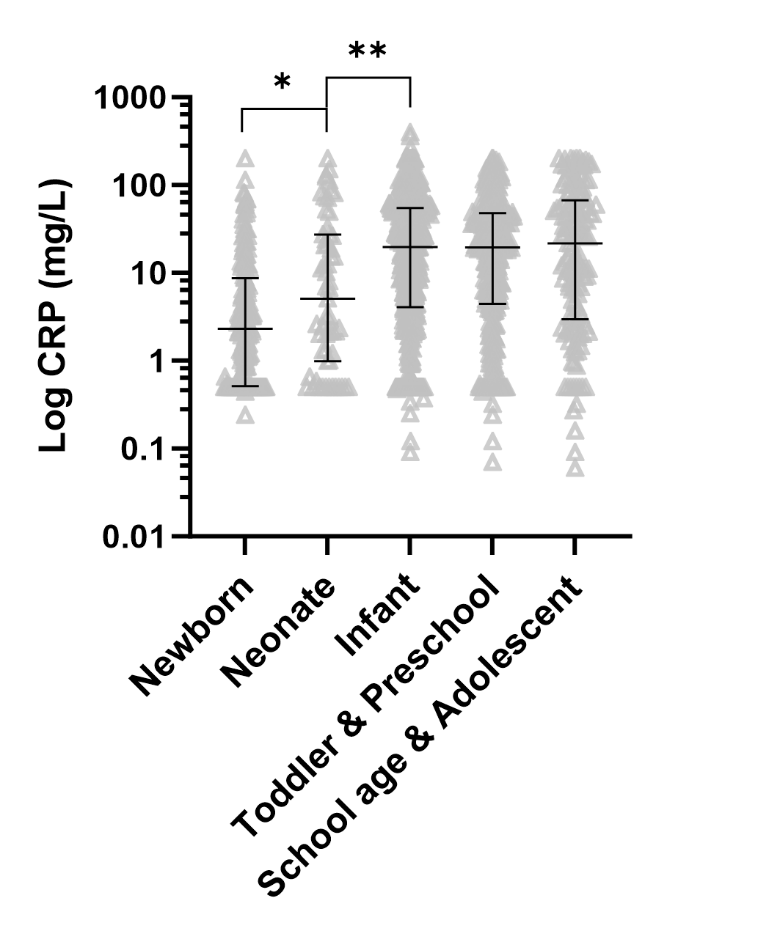


**Figure S1 The distribution of individual CRP levels across age groups.**

**All individual CRP values were presented in the dot plot. A notable age-dependent increase in median CRP levels was observed. Newborns and neonates exhibit the lowest values, followed by a steep increase through infancy, and a gradual plateau. The central line indicates the median, the whisker indicates the interquartile range. (* < 0.05, ** < 0.01)**

**Table S2 CRP levels of preterm and term patients in the newborn and neonate groups.**

|  | **Preterm** | **Term** | ***P*** |
| --- | --- | --- | --- |
| **Newborn** | 1.41 (0.50 ~ 5.85), N=79 | 2.91 (1.00 ~ 12.13), N=96 | **0.019** |
| **Neonate** | 3.20 (0.50 ~ 45.01), N=17 | 2.83 (0.91 ~ 24.38), N=38 | 0.920 |
| ***P*** | 0.107 | 0.543 |  |

Raw *P* values are shown. Bonferroni correction was applied for the two comparisons (preterm vs. term within the newborn group and within the neonate group); Raw *P* < 0.025 (α=0.05/2) was considered statistically significant.

**Table S3 Multivariable linear regression analysis of factors associated with Log^CRP^ levels in newborns.**

| **Variable** | **β**  **(Unstandardized)** | **95% CI** | **β**  **(Standardized)** | ***P*** |
| --- | --- | --- | --- | --- |
| **(Constant)** | -0.255 | -1.596 ~ 1.086 |  | 0.708 |
| **Gestational age**  **(Month)** | 0.010 | -0.037 ~ 0.057 | 0.053 | 0.678 |
| **Birth weight**  **(Gram)** | 0.000 | 0.000 ~ 0.000 | 0.188 | 0.148 |
| **Sex**  **(Female vs. Male)** | -0.107 | -0.307 ~ 0.093 | -0.078 | 0.291 |
| **Invasive ventilation**  **(No vs. Yes)** | 0.183 | -0.073 ~ 0.439 | 0.138 | 0.159 |
| **Vasoactive drug**  **(No vs. Yes)** | -0.236 | -0.490 ~ 0.019 | -0.178 | 0.069 |

Dependent variable: Log^CRP^. R² = 0.082, adjusted R² =0.054. The regression included 175 patients.

**Table S4 Days from symptom onset to admission blood sampling by age group.**

| **Age group** | **N (available / total)** | **Median (IQR), days** | ***P*** |
| --- | --- | --- | --- |
| **Newborn** | 186 / 186 | 2.00 (1.00 ~ 4.00) |  |
| **Neonate** | 62 / 62 | 1.00 (1.00 ~ 3.00) | **0.037** |
| **Infant** | 352 / 485 | 3.00 (1.25 ~ 5.75) | **< 0.001** |
| **Toddler and Preschool** | 231 / 351 | 3.00 (2.00 ~ 6.00) | **< 0.001** |
| **School age and Adolescent** | 107 / 144 | 4.00 (2.00 ~ 7.00) | **< 0.001** |

*P* values were calculated using Mann-Whitney U test comparing each group with Newborn. The *P* values are presented for descriptive purposes and were not adjusted for multiple comparisons.

**Table S5 Multivariable linear regression analysis of factors associated with Log^CRP^ in term newborn/neonate and term infant groups.**

| **Variable** | **β**  **(Unstandardized)** | **95% CI** | **β**  **(Standardized)** | ***P*** |
| --- | --- | --- | --- | --- |
| **(Constant)** | 0.381 | 0.043 ~ 0.718 |  | **0.027** |
| **Age group**  **(Term newborn and neonate**  **vs.**  **Term infant)** | 0.455 | 0.281 ~ 0.629 | 0.256 | **< 0.001** |
| **Sex**  **(Female vs. Male)** | -0.083 | -0.224 ~ 0.058 | -0.051 | 0.245 |
| **Invasive ventilation**  **(No vs. Yes)** | -0.246 | -0.524 ~ 0.031 | -0.112 | 0.082 |
| **Vasoactive drug**  **(No vs. Yes)** | -0.126 | -0.417 ~ 0.164 | -0.053 | 0.394 |
| **Onset‑to‑sampling Interval**  **(Days)** | -0.019 | -0.037 ~ -0.002 | -0.100 | **0.025** |

Dependent variable: Log^CRP^. R² = 0.129, adjusted R² = 0.120. The regression included 468 patients.

**Table S6 Comparison of disease severity indicators between proven and suspected infection subgroups.**

| **vital signs** | **Age groups** | **Proven infection**  **(No / Yes)*** | **Suspected infection**  **(No / Yes)** | **Risk difference (95% CI)**** | ***P*** |
| --- | --- | --- | --- | --- | --- |
| **Invasive ventilation** | Term newborn and neonate | 13 / 14 | 64 / 45 | 10.6% (-11.7 ~ 32.8) | 0.387 |
|  | Term infant | 106 / 9 | 346 / 8 | 5.5% (0 ~ 11.1) | **0.01** |
|  | Toddler and Preschool | 80 / 0 | 264 / 0 | 0% (not estimable) | / |
|  | School age and Adolescent | 44 / 1 | 97 / 0 | 2.2% (-2.3 ~ 6.8) | 0.317 |
| **Vasoactive infusion** | Term newborn and neonate | 16 / 11 | 72 / 37 | 6.8% (-13.5 ~ 27.1) | 0.509 |
|  | Term infant | 109 / 6 | 345 / 9 | 2.7% (-1.2 ~ 6.6) | 0.217 |
|  | Toddler and Preschool | 79 / 1 | 264 / 0 | 1.3% (-1.5 ~ 4.0) | 0.233 |
|  | School age and Adolescent | 45 / 0 | 97 / 0 | 0% (not estimable) | / |

*, The number of patients receiving invasive ventilation or vasoactive infusion. **, Risk difference (proven-suspected) with 95% confidence intervals was analyzed by Newcombe‑Wilson method.

“/” indicates that the test was not performed because of zero events in both subgroups.

Raw *P* values are shown. Raw *P* < 0.0125 (Bonferroni correction, α=0.05/4) was considered statistically significant.

**Table S7** **Comparison of vital signs between proven and suspected infection subgroups.**

| **vital signs** | **Age groups** | **Proven infection** | **Suspected infection** | **Median difference (95% CI)** | ***P*** |
| --- | --- | --- | --- | --- | --- |
| **Pulse rate,**  **Beats/minute** | Term newborn and neonate | 142 (131 ~ 155)  N=27 | 145 (140 ~ 150)  N=109 | -2 (-8 ~ 5) | 0.539 |
|  | Term infant | 130 (122 ~ 140)  N=115 | 128 (120 ~ 135)  N=354 | 4 (1 ~ 6) | **0.005** |
|  | Toddler and Preschool | 110 (101 ~ 118)  N=80 | 105 (98 ~ 116)  N=264 | 2 (0 ~ 6) | 0.077 |
|  | School age and Adolescent | 95 (90 ~ 100)  N=45 | 94 (88 ~ 99)  N=97 | 2 (-2 ~ 4) | 0.411 |
| **Respiratory rate, Breaths/minute** | Term newborn and neonate | 50 (41 ~ 64)  N=27 | 50 (46 ~ 60)  N=109 | 0 (-5 ~ 4) | 0.716 |
|  | Term infant | 33 (28 ~ 40)  N=115 | 32 (28 ~ 36)  N=354 | 1 (0 ~ 3) | 0.099 |
|  | Toddler and Preschool | 26 (24 ~ 28)  N=80 | 25 (23 ~ 28)  N=264 | 1 (0 ~ 2) | 0.132 |
|  | School age and Adolescent | 23 (21 ~ 25)  N=45 | 23 (22 ~ 24)  N=97 | 0 (-1 ~ 1) | 0.837 |
| **Mean arterial pressure, mm Hg** | Term newborn and neonate | 53 (50 ~ 59)  N=27 | 52 (48 ~ 57)  N=109 | 2 (-1 ~ 6) | 0.187 |
|  | Term infant | 60 (59 ~ 63)  N=115 | 61 (59 ~ 64)  N=354 | 0 (-1 ~ 1) | 0.955 |
|  | Toddler and Preschool | 68 (64 ~ 70)  N=80 | 67 (64 ~ 70)  N=264 | 0 (-1 ~ 1) | 0.769 |
|  | School age and Adolescent | 73 (70 ~ 76)  N=45 | 71 (70 ~ 73)  N=97 | 1 (0 ~ 3) | 0.091 |

Raw *P* values are shown. Raw *P* < 0.0125 (Bonferroni correction, α=0.05/4) was considered statistically significant. Median difference was analyzed by Hodges-Lehmann method.

**Table S8 CRP levels in proven and suspected infection subgroups.**

| **Age groups** | **Proven infection** | **Suspected infection** |
| --- | --- | --- |
| **Term newborn and neonate** | 5.38 (1.89 ~ 31.70)  N=26 | 2.46 (0.85 ~ 11.16)  N=107 |
| **Term infant** | 24.46 (4.53 ~ 69.58)  N=111 | 18.06 (4.03 ~ 53.50)  N=346 |
| **Toddler and Preschool** | 30.08 (5.00 ~ 50.51)  N=78 | 18.56 (3.98 ~ 45.34)  N=258 |
| **School age and Adolescent** | 46.67 (2.72 ~ 112.53)  N=41 | 15.00 (3.18 ~ 53.03)  N=96 |
| ***P*** | **0.036** | **< 0.001** |

*P* values were calculated using Kruskal-Wallis test.

**Table S9 Pairwise comparisons of CRP levels across age groups in proven and suspected infection subgroups.**

| **Comparison** | **Proven infection** | | **Suspected infection** | |
| --- | --- | --- | --- | --- |
|  | **Median difference (95% CI)** | **Adjusted *P*** | **Median difference (95% CI)** | **Adjusted *P*** |
| **Term infant**  **vs.**  **Term newborn and neonate** | 9.57 (1.03 ~ 24.93) | 0.126 | 11.64 (6.72 ~ 17.49) | **< 0.001** |
| **Toddler and Preschool**  **vs.**  **Term newborn and neonate** | 8.20 (0.43 ~ 26.75) | 0.283 | 11.42 (6.29 ~ 16.55) | **< 0.001** |
| **School age and Adolescent vs.**  **Term newborn and neonate** | 30.52 (1.32 ~ 55.00) | **0.024** | 9.96 (5.69 ~ 17.71) | **< 0.001** |
| **Toddler and Preschool**  **vs.**  **Term infant** | 0.71 (-5.12 ~ 8.54) | 1.000 | 0.63 (-1.38 ~ 3.54) | 1.000 |
| **School age and Adolescent**  **vs.**  **Term infant** | 7.53 (-2.68 ~ 34.28) | 1.000 | 0.00 (-3.78 ~ 3.92) | 1.000 |
| **School age and Adolescent**  **vs.**  **Toddler and Preschool** | 11.07 (-1.16 ~ 38.93) | 0.940 | 0.57 (-3.27 ~ 5.19) | 1.000 |

Median difference was analyzed by Hodges-Lehmann method. *P* values adjusted by Bonferroni correction are shown. Adjusted *P* < 0.05 was considered statistically significant.

**Table S10 CRP levels in patients with different pathogen categories across age groups.**

| **Age group** | **Bacteria** | **Fungus** | **Virus** | **Mycoplasma/ Chlamydia/Ureaplasma** | **Polymicrobial infection** |
| --- | --- | --- | --- | --- | --- |
| **Newborn** | 8.87 (1.69 ~ 47.38)  N=28 | 0.50 (0.50 ~ 12.19)  N=5 | / | 3.64; 5.56  N=2 | 3.32; 5.42  N=2 |
| **Neonate** | 12.40 (2.14 ~ 71.25)  N=15 | 3.20; 27.15  N=2 | 0.50  N=1 | 0.50; 1.30  N=2 | 0.50; 2.00  N=2 |
| **Infant** | 31.70 (8.19 ~ 80.00)  N=81 | 5.81; 12.41; 113.71  N=3 | 7.20 (3.25 ~ 19.25)  N=16 | 0.50; 10.06; 19.63; 157.84  N=4 | 12.03 (0.96 ~ 73.73)  N=11 |
| **Toddler and Preschool** | 34.25 (8.43 ~ 57.33)  N=56 | 2.01  N=1 | 6.27 (3.04 ~ 45.00)  N=17 | 8.68; 30.54; 34.78  N=3 | 1.92  N=1 |
| **School age and Adolescent** | 60.99 (4.65 ~ 155.39)  N=28 | 43.41; 112.68  N=2 | 1.82 (0.50 ~ 46.24)  N=5 | 2.03; 4.03; 10.26; 170.00  N=4 | 11.18; 55.50  N=2 |

Data are presented as median (IQR) for subgroups with ≥ 5 cases, and as individual raw values for subgroups with < 5 cases. N denotes the number of patients in each subgroup. Due to small sample sizes in several subgroups, formal statistical comparisons were not performed; data should be interpreted as descriptive only. “/” indicates no cases in that category.

**Table S11 Percentile table of CRP levels for each age group.**

| **Percentile** | **Newborn**  **(N=181)** | **Neonate**  **(N=59)** | **Infant**  **(N=471)** | **Toddler and Preschool**  **(N=341)** | **School age and Adolescent**  **(N=139)** |
| --- | --- | --- | --- | --- | --- |
| **5** **th** | 0.50 | 0.50 | 0.50 | 0.50 | 0.50 |
| **10 th** | 0.50 | 0.50 | 0.61 | 0.55 | 0.87 |
| **25 th** | 0.52 | 0.99 | 4.06 | 4.43 | 2.98 |
| **50 th** | 2.32 | 5.09 | 19.61 | 19.43 | 21.60 |
| **75 th** | 8.74 | 27.15 | 54.97 | 47.86 | 66.98 |
| **90 th** | 30.38 | 86.82 | 102.96 | 101.20 | 165.17 |
| **95 th** | 58.13 | 130.29 | 152.45 | 135.40 | 190.97 |

N denotes the number of available patients in each age group.
